# Bergenin, Acting as an Agonist of PPARγ, Ameliorates Experimental Colitis in Mice through Improving Expression of SIRT1, and Therefore Inhibiting NF-κB-Mediated Macrophage Activation

**DOI:** 10.3389/fphar.2017.00981

**Published:** 2018-01-12

**Authors:** Kai Wang, Yun-fan Li, Qi Lv, Xi-ming Li, Yue Dai, Zhi-feng Wei

**Affiliations:** Department of Pharmacology of Chinese Materia Medica, China Pharmaceutical University, Nanjing, China

**Keywords:** bergenin, ulcerative colitis, pro-inflammatory cytokines, PPARγ, SIRT1

## Abstract

Bergenin, isolated from the herb of *Saxifraga stolonifera* Curt. (Hu-Er-Cao), has anti-inflammatory, antitussive and wound healing activities. The aim of the present study was to identify the effect of bergenin on experimental colitis, and explored the related mechanisms. Our results showed that oral administration of bergenin remarkably alleviated disease symptoms of mice with dextran sulfate sodium (DSS)-induced colitis, evidenced by reduced DAI scores, shortening of colon length, MPO activity and pathologic abnormalities in colons. Bergenin obviously inhibited the mRNA and protein expressions of IL-6 and TNF-α in colon tissues, but not that of mucosal barrier-associated proteins occludin, *E*-cadherin and MUC-2. *In vitro*, bergenin significantly inhibited the expressions of IL-6 and TNF-α as well as nuclear translocation and DNA binding activity of NF-κB-p65 in lipopolysaccharide (LPS)-stimulated peritoneal macrophages and RAW264.7 cells, which was almost reversed by addition of PPARγ antagonist GW9662 and siPPARγ. Subsequently, bergenin was identified as a PPARγ agonist. It could enter into macrophages, bind with PPARγ, promote nuclear translocation and transcriptional activity of PPARγ, and increase mRNA expressions of CD36, LPL and ap2. In addition, bergenin significantly up-regulated expression of SIRT1, inhibited acetylation of NF-κB-p65 and increased association NF-κB-p65 and IκBα. Finally, the correlation between activation of PPARγ and attenuation of colitis, inhibition of IL-6 and TNF-α expressions, NF-κB-p65 acetylation and nuclear translocation, and up-regulation of SIRT1 expression by bergenin was validated in mice with DSS-induced colitis and/or LPS-stimulated macrophages. In summary, bergenin could ameliorate colitis in mice through inhibiting the activation of macrophages *via* regulating PPARγ/SIRT1/NF-κB-p65 pathway. The findings can provide evidence for the further development of bergenin as an anti-UC drug, and offer a paradigm for the recognization of anti-UC mechanisms of compound with similar structure occurring in traditional Chinese medicines.

## Introduction

Ulcerative colitis (UC) is a chronic inflammatory disease, which mainly occurs at the colon site and spreads from rectum to proximal colon and even ileum (Baumgart and Sandborn, 2007). It has been reported with high incidences in many regions: Northern Europe (24.3 per 100000), Canada (19.2 per 100000), and Australia (17.4 per 100000) (Ungaro et al., 2017). The pathogenesis of UC remains elusive, but destruction of gastrointestinal mucosa barrier and persistent inflammation has been demonstrated to occupy important positions (Ananthakrishnan, 2015). The epithelium of mucosa plays an essential role in maintaining balance of intestinal ecosystem. In UC patients, the intestinal mucosal barrier presents varying degrees damage, permeability of intestinal epithelial cells rises, and the ability of enteric pathogens and their toxins to pass through and enter the epithelial layer increases. In addition, data indicate that many activated macrophages exist in colonic mucosa, secret inflammatory cytokines such as IL-1β, IL-6 and TNF-α, and control persistent inflammation. In the sera and colonic tissues of UC patients, the levels of IL-1β, IL-6 and TNF-α were significantly higher than those of normal subjects, while down-regulation of them could prevent UC (Galli et al., 2011; Roberts-Thomson et al., 2011; Groeger et al., 2013). Clinically, tocilizumab, the IL-6 receptor (IL-6R) antibody, has been used to treat UC (Nishimoto et al., 2008; Wu et al., 2015); TNF-α antibody is also effective for UC management, especially for severe disease, and its bio-similars are confirmed with well safety and efficacy (Sofia and Rubin, 2016; Komaki et al., 2017). Therefore, repairment of mucosal barrier and inhibition of inflammation are effective strategies for UC treatment.

PPARγ, an important member of nuclear receptor family, can be tracked in multiple kinds of cells, including intestinal epithelial cells, macrophages, lymphocytes, etc., and participates in regulation of inflammation and mucosal damage in UC lesions (Bertin et al., 2013). Ligand-binding domain of PPARγ interacts with many structurally different small molecules and exerts action. On the one hand, PPARγ directly binds to peroxisome proliferator response elements (PPREs) and promotes the expressions of target genes; on the other hand, the activation of PPARγ regulates the expressions of multiple pro-inflammatory cytokines and barrier function-associated proteins by interfering with the activity of transcription factors, such as NF-κB and AP-1 (Bonfield et al., 2008). In colonic epithelium of UC patients, PPARγ expression was down-regulated (Dubuquoy et al., 2003); PPARγ agonist rosiglitazone could treat moderate UC (Lewis et al., 2008; Pedersen and Brynskov, 2010); 5-aminosalicylic acid (5-ASA), a traditional UC therapeutic drug, ameliorated the symptoms of wild-type colitis mice, but was ineffective in PPARγ^+/-^ heterozygous mice (Rousseaux et al., 2005).

Bergenia purpurascens, a traditional Chinese medicine, possesses abilities of anti-inflammation and anti-diarrhea, and clinically used for the treatment of diarrhea, dysentery and other gut-associated diseases. Bergenin is the major bioactive ingredient in the herb drug. It could inhibit collagen-induced arthritis in mice by reducing serum levels of IL-2, TNF-α and IL-6, and down-regulate lipopolysaccharide (LPS)-induced activation of THP-1 cells by decreasing levels of NF-κB and IKKβ (Jain et al., 2014). In addition, bergenin prevented the production of Th1 cytokines (IL-2, IFN-γ and TNF-α) while up-regulated Th2 cytokines (IL-4 and IL-5) in the peripheral blood of mice with adjuvant-induced arthritis (Nazir et al., 2007); bergenin reduced the expressions of NO, TNF-α, IL-1β and IL-6 in mammary glands of mice with LPS-induced mastitis by inhibiting the activation of NF-κB and MAPKs signaling pathways (Gao et al., 2015); bergenin had a therapeutic effect on LPS-induced acute lung injury by inhibiting NF-κB activation (Yang et al., 2017). In addition, bergenin belongs to isocoumarins, sparstolonin B, with similar structure, could activate PPARγ (Wang et al., 2015). In this study, dextran sulfate sodium (DSS)-induced colitis was established in mice to evaluate the effect of bergenin, and the underlying mechanisms were explored from the angle of PPARγ-dependence.

## Materials and Methods

### Chemicals and Reagents

Bergenin (purity > 98%) was purchased from Nanjing JingZhu Biological Technology Co., Ltd. (Nanjing, China); dextran sulfate sodium (DSS, molecular weight 36–50 kDa) was purchased from MP Biomedical (OH, United States); 5-aminosalicylic acid (5-ASA) sustained release granules were purchased from Ipsen Pharma (Houdan, France); Rosiglitazone was purchased from Ampere Reagent Co., Ltd. (Shenzhen, China); GW9662 (a PPAR-γ antagonist) and LPS were purchased from Sigma Chemical Co., Ltd. (St. Louis, MO, United States); myeloperoxidase (MPO) activity kit was purchased from JianCheng Bioengineering Institute (Nanjing, China); TNF-α and IL-6 enzyme-linked immunosorbent assay (ELISA) kits were purchased from Dakewe Biotech Co., Ltd. (Shenzhen, China); siPPARγ was purchased from RiboBio Co. (Guangzhou, China); enhanced chemiluminescent (ECL) plus reagent kit was purchased from DiZhao Biotech Co., Ltd (Shanghai, China); LanthaScreen^®^ TR-FRET PPAR-γ competitive binding assay Kit was purchased from Thermo Fisher Scientific Co. (Waltham, MA, United States); TRIzol reagent was purchased from Invitrogen (Carlsbad, CA, United States); HiScriptTM reverse transcriptase system and SYBR@ green master mix were purchased from Vazyme Biotech Co., Ltd. (Nanjing, China); Luciferase Reporter Gene Assay Kit was purchased from Beyotime (Shanghai, China). The antibodies used in western blotting (WB), immunofluorescence (IF) and immunoprecipitation (IP) assays are listed in **Table 1**.

**Table 1:** Antibodies used in western blotting (WB), immunofluorescence (IF) and immunoprecipitation (IP) assays.

Antibodies	Brand	Catalog no.	Applications
β-actin Rabbit Polyclonal	Shenyang wanlei	WL01372	WB: 1: 1000
Lamin B1 Rabbit Polyclonal	Shenyang wanlei	WL01775	WB: 1: 500
IKKβ Rabbit Polyclonal	Shenyang wanlei	WL01900	WB: 1: 500
p-NFκB-p65 Rabbit Polyclonal	Shenyang wanlei	WL02169	WB: 1:500
SIRT1 Rabbit Polyclonal	Shenyang wanlei	WL00599	WB: 1: 500
PPAR-γ Rabbit monoclonal	Epitopmics	EP4394(N)	WB: 1: 1000
p-IKKβ Rabbit Polyclonal	Bioworld	O14920	WB: 1: 1000
p-IκBα Rabbit Polyclonal	Bioworld	P25963	WB: 1: 1000
IkBα Rabbit Polyclonal	Bioworld	BS6227	WB: 1: 1000; IP: 1: 100
NF-κB-p65 Rabbit Polyclonal	Proteintech	10745-1-AP	WB: 1: 2000; IP: 1: 200; IF: 1: 200
Ace-p65 Rabbit Polyclonal	Keygen Biotech	KGYK0018-6	WB: 1: 1000; IF: 1: 100

### Animals

Female C57BL/6 mice, 6–8-weeks-old, were obtained from the Comparative Medicine of Yangzhou University (Yangzhou, China). All animals were housed under a 12 h light/12 h dark cycle (lights on from 7 am to 7 pm) with controlled room temperature (about 25°C) and humidity (50–65%) in the cages (290 mm × 178 mm × 160 mm), and allowed *ad libitum* access to a diet of standard laboratory chow and water. The efforts were made to minimize the animals’ suffering and to reduce the number of animals used. This study was carried out in accordance with the current ethical regulations for institutional animal care and use in China Pharmaceutical University. The protocol was approved by the Animal Ethics Committee of China Pharmaceutical University.

### Induction of UC and Administration of Bergenin

The colitis was induced in C57BL/6 mice by oral administration of 2.5% DSS in drinking water for 7 days. Mice were then provided with normal drinking water for another 3 days. They were randomly divided into the following groups: (a) Normal group, DSS group, bergenin (20, 50 mg/kg) group and 5-ASA (100 mg/kg) group; (b) Normal group, DSS group, GW9662 (1 mg/kg) group, bergenin (50 mg/kg) group, GW9662+bergenin group, 5-ASA (100 mg/kg) group and rosiglitazone (20 mg/kg) group. Bergenin, rosiglitazone and 5-ASA were orally administered by gastric gavage from day 1 to 10; GW9662 was intraperitoneally administered from day 1 to 10. Bergenin was dissolved in physiological saline; rosiglitazone and 5-ASA were suspended in 0.5% CMC-Na; GW9662 was dissolved in DMSO/physiological saline 1: 10. The volume of oral and intraperitoneal administration was 0.1 mL/10 g.

On day 10, mice were sacrificed with ether anesthesia, and the colons were gathered and photographed. Then, the length of colons was measured and recorded, and the full colons were stored at -80°C in refrigerator. The MPO activity, quantitative-polymerase chain reaction (Q-PCR) and ELISA were done on complete colon tissues.

### Disease Activity Index (DAI)

The DAI scores were calculated according to loss of body weight, stool consistency and gross bleeding, and exhibited as the mean value of the following three parameters: (a) body weight loss: 0 = none; 1 = 1–5%; 2 = 5–10%; 3 = 10–15%; 4 = over 15%; (b) stool consistency: 0 = normal; 2 = loose stools; 4 = diarrhea; (c) gross bleeding: 0 = normal; 2 = hemoccult; 4 = gross bleeding.

### MPO Activity

The activity of MPO in colons was measured using commercial kits according to the manufacturer’s instructions from JianCheng Bioengineering Institute (Nanjing, China).

### Histological Evaluation

The distal end of colons was fixed in 4% formaldehyde, embedded in paraffin, sectioned, and stained with hematoxylin and eosin according to standard protocols. The histological score was graded as follows: (a) inflammation severity: 0 = none; 1 = slight; 2 = moderate; 3 = severe; 4 = very severe; (b) lesion depth: 0 = none; 1 = mucosal layer; 2 = submucosal layer; 3 = muscle layer; 4 = transmural; (c) crypt damage: 0 = none; 1 = basal 1/3 damaged; 2 = basal 2/3 damaged; 3 = only surface epithelium intact; 4 = entire crypt and epithelium lost; lesion range: 1 = 1–25%; 2 = 26–50%; 3 = 51–75%; 4 = 76–100%.

### Q-PCR Assay

Total RNA of colons or cells was extracted by using TRIzol extraction reagent, and RNA purity and concentration were determined by measuring the absorbance at 260 and 280 nm. Then, the total RNA was reversely transcribed into cDNA and subjected to Q-PCR, which was performed with the HiScript^™^ reverse transcriptase system and SYBR@ green master mix (Vazyme; Nanjing, China). The threshold cycle numbers were obtained by MyiQ2 Detection System (Bio-Rad Laboratories, Hercules, CA, United States), and primer sequences used were listed in **Table 2**. The expression of each gene was normalized to GAPDH, and calculated according to the 2^-ΔΔ^Ct method (Xu et al., 2017).

**Table 2:** Primers used in quantitative-polymerase chain reaction (Q-PCR).

Primers	Accession no.		Sequence (5′→3′)
IL-1β	NM_008361.4	Forward	AGTTGACGGACCCCAAAAG
		Reverse	CTTCTCCACAGCCACAATGA
IL-6	NM_031168.2	Forward	CGGAGAGGAGACTTCACAGAG
		Reverse	ATTTCCACGATTTCCCAGAG
TNF-α	NM_013693.3	Forward	AGGCACTCCCCCAAAAGAT
		Reverse	CAGTAGACAGAAGAGCGTGGTG
E-cadherin	NM_009864.3	Forward	GAGGAGAACGGTGGTCAAAG
		Reverse	GCTGGCTCAAATCAAAGTCC
Occludin	NM_008756.2	Forward	TTCCTCTGACCTTGAGTGTGG
		Reverse	CTCTTGCCCTTTCCTGCTTT
MUC-2	NM_023566.3	Forward	CTCGGTCTCCAACATCACCT
		Reverse	GAGCAAGGGACTCTGGTCTG
CD36	NM_001159556.1	Forward	CCCTCCAGAATCCAGACAAC
		Reverse	CACAGGCTTTCCTTCTTTGC
LPL	NM_008509.2	Forward	ACACATTTACCAGGGGGTCA
		Reverse	AATCACACGGATGGCTTCTC
aP2	NM_024406.3	Forward	AAATCACCGCAGACGACAG
		Reverse	TCATAACACATTCCACCACCA
GAPDH	NM_008084.3	Forward	GACATTTGAGAAGGGCCACAT
		Reverse	CAAAGAGGTCCAAAACAATCG

### Enzyme-Linked Immunosorbent Assay

The colon tissues were homogenated with PBS and centrifuged at 3000 rpm for 20 min, and the supernatants were collected. Then, the protein levels of TNF-α and IL-6 were determined by using ELISA kits according to the manufacturer’s instructions.

### Cell Culture and Viability Assay

RAW264.7 cells were purchased from China Center for Type Culture Collection (Wuhan, China) and maintained in Dulbecco’s modified Eagle’s medium (DMEM) containing 10% fetal bovine serum (FBS) in 5% CO_2_ at 37°C.

Peritoneal macrophages isolation and culture: mice were intraperitoneally injected with PBS, and the lavage fluid was obtained from the peritoneal cavity and centrifuged at 1000 rpm for 5 min. Then, the precipitate was collected, washed with PBS for twice, and suspended in RPMI-1640 medium containing 10% FBS. Subsequently, the cells were seeded into 6-well plates and cultured with RPMI-1640 medium containing 10% FBS for 4 h. The non-adherent cells were removed, and the adherent cells were used as macrophages for the following experiments.

The viability of RAW264.7 cells and peritoneal macrophages was evaluated by using MTT assay. Briefly, cells were seeded into 96-well plates and incubated with bergenin (0, 1, 3, 10, 30 μM) for 20 h. Then, 20 μL of MTT solution (5 mg/mL in PBS) was added into each well, and cells were continuously cultured for another 4 h. Last, the supernatants were removed, and DMSO was added into each well to dissolve the formazan crystals. The absorbance was determined at 570 nm.

### Western Blotting Assay

The total proteins of colons or cells were prepared by using NP40 buffer (Beyotime, Nanjing, China), and cytoplasmic and nuclear proteins were isolated by using nuclear and cytoplasmic protein extraction kits (JianCheng Bioengineering Institute, Nanjing, China). Then, samples were stored at -80°C for western blot assay: protein lysates were separated by 10% SDS-PAGE and electrotransferred to PVDF membrane. The membrane was blocked with 5% non-fat milk for 2 h at room temperature, and incubated with different primary antibodies at 4°C for overnight. Then, membranes were incubated with IRDye-conjugated secondary antibody for 2 h at room temperature. Finally, signals were detected by using enhanced chemiluminescence (Pierce, Holmdel, NJ, United States).

### Electrophoretic Mobility Shift Assay (EMSA)

For detecting the DNA binding activity of NF-κB, the EMSA assay was performed by using a commercial kit (Pierce, Rockford, IL, United States). Briefly, RAW264.7 cells (4 × 10^5^ cells/mL) were seeded into 6-well plates and incubated with bergenin for 24 h. Biotin-labeled NF-κB-specific oligonucleotides were prepared as the labeled probe according to the manufacturer’s instructions; the nuclear extracts were incubated with poly (dI-dC), labeled probe and binding buffer at 25°C for 10 min. Then, reaction mixtures were separated with 5% non-denatured polyacrylamide gels at 1mA/cm at 4°C for 1.5 h and transferred to a PVDF membrane. The biotin end-labeled DNA was tested with a streptavidin-HRP conjugate and a chemiluminescent substrate. Finally, membranes were detected with X-ray film and analyzed with the Quantity One software (BD Biosciences, San Jose, CA, United States) (Lee et al., 2012).

### High Performance Liquid Chromatography (HPLC) Assay

RAW264.7 cells (4 × 10^5^ cells/mL) were seeded into 6-well plates and incubated with bergenin (30 μM) for 0.5, 1, 2, 4, 8, 16, and 24 h at 37°C under 5% CO_2_ condition, and supernatants were removed. The cells were washed with PBS for three times, and disrupted by multigelation for three times (-80°C for 1 h and 37°C for 5 min). Then, they were centrifuged to further remove the cell membranes. The supernatant was collected, and HPLC assay was performed.

Then, endoctoysis of bergenin in RAW264.7 cells was determined by using HPLC assay. The chromatographic separation was accessed with a Hedera ODS-2 C18 column (150 mm × 2.1 mm, 5 μm; Hanbon, Jiangsu, China) at 40°C. The mobile phase consisted of solvent A (0.1% formic acid in water) and solvent B (acetonitrile) (72: 28, v/v) at a flow rate of 1 mL/min. The detection wavelength was set at 275 nm.

### siRNA Transfection

Three pairs of PPAR-γ siRNA and one pair of Ncontrol siRNA were designed and synthesized by RiboBio Co. (RiboBio, Guangzhou, China). RAW264.7 cells (4 × 10^5^ cells/mL) were seeded into 6-well plates and cultured for 24 h, and transfected with siPPAR-γ or siNcontrol for 5 h by using lipofectamine 2000 (Invitrogen, Carlsbad, CA, United States) according to the manufacturer’s instructions. Then, the supernatants were removed and replaced with fresh medium. Subsequently, cells were cultured for 24 h, and used for subsequent experiments. Total mRNA was harvested for monitoring transfection efficiency by using Q-PCR assay.

### Competition Binding Assay

The LanthaScreen TR-FRET PPARγ competitive binding assay was performed according to the manufacturer’s protocol. Bergenin or rosiglitazone was cultured with glutathione s-transferase (GST)-fused human PPARγ-ligand binding domain (LBD), terbium-labeled anti-GST antibody and a fluorescently labeled PPAR ligand for 3 h in the dark at room temperature. The FRET signal was valued by excitation at 340 nm and emission at 520 nm for fluorescein and 495 nm for terbium. The ability of binding to the PPARγ-LBD was measured by the ratio of emission signal at 520 and 495 nm (Nevin et al., 2012; Weidner et al., 2012).

### Luciferase Assay

RAW264.7 cells (4 × 10^5^ cells/mL) were seeded into 24 well plates, and co-transfected with PPRE-REPO. A PPRE-driven luciferase reporter plasmid was applied for examining the specific activation of PPARγ binding to the PPARγ response element (PPRE). 24 h later, the supernatants were removed. Then, the cells were incubated with bergenin and rosiglitazone for another 24 h, and washed and lysed. The supernatants were collected. Luciferase activity was measured by using a luciferase assay system and a multimode reader according to the manufacturer’s instructions.

### Immunofluorescence Assay

RAW264.7 cells (4 × 10^5^ cells/mL) were seeded on coverslips, and fixed in 4% paraformaldehyde (PFA) for 20 min at room temperature. After washing with PBS, cells were permeabilized with 0.25% Triton X-100 for 1 h, and blocked with 5% BSA for 2 h. Then, cells were immune-stained with monoclonal antibody at 4°C for overnight, and incubated with Alexa Fluor-labeled secondary antibody (1: 100) for 1 h. Finally, the images were gained with a fluorescence microscope.

### Co-immunoprecipitation Assay

RAW264.7 cells (4 × 10^5^ cells/mL) were seeded into cell culture flasks, lysed with NP40 buffer for 10 min, and centrifuged at 12, 000 rpm for 10 min. Then, the soluble fractions were used in the following experiments. The cell lysates were incubated with 2 μg IκBα antibody for overnight, and followed by incubation of 20 μL protein A/G agarose for another 4 h at 4°C. Then, immunoprecipitates proteins were washed with NP40 buffer, and separated by SDS-PAGE as described in western blotting assay.

### Statistical Analysis

Statistical analysis was performed with SPSS statistical software (SPSS, Chicago, IL, United States), and data were expressed as means ± SEM. The mean differences between two groups were compared by *t*-test; the mean differences between multiple groups were compared by one-way ANOVA and Fisher’s Least Significant Difference (LSD) test. A value of *P* less than 0.05 (*P* < 0.05) was accepted as a significant difference.

## Results

### Effect of Bergenin on DSS-Induced Colitis in Mice

**FIGURE 1:**
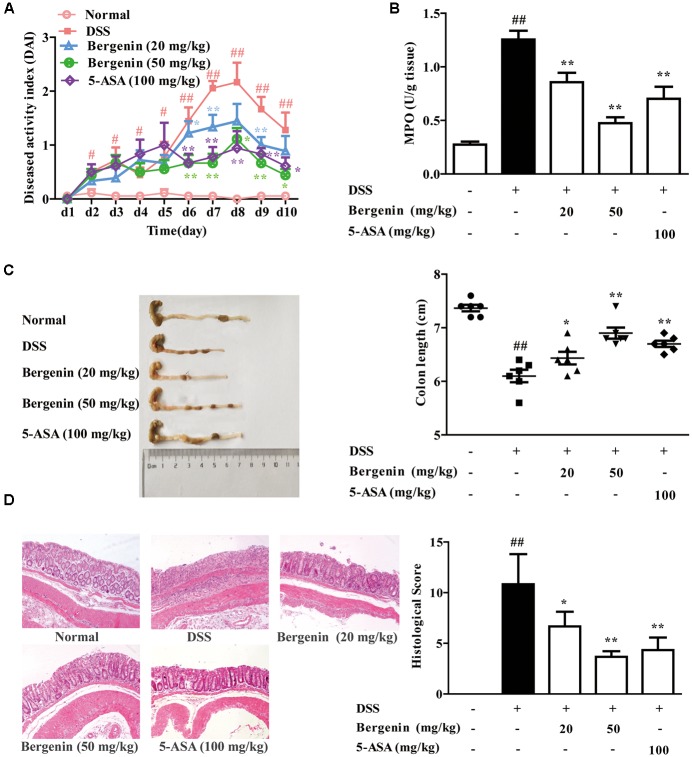
Effect of bergenin on DSS-induced colitis in mice. Mice were treated with 2.5% DSS in drinking water for 7 days, provided with drinking water for another 3 days. Bergenin (20, 50 mg/kg; i.g.) and 5-ASA (100 mg/kg; i.g.) were administrated daily for consecutive 10 days. **(A)** Disease activity index (DAI). **(B)** Myeloperoxidase (MPO) activity in colons. **(C)** Length of colons. **(D)** Representative images showing colon pathologic abnormalities with hematoxylin and eosin (H&E) staining. Histological scores were analyzed from H&E staining. Data were presented as the means ± SEM (*n* = 6). ^##^*p* < 0.01 vs. the group without any treatment; ^∗^*p* < 0.05 and ^∗∗^*p* < 0.01 vs. DSS group.

UC was induced in female C57BL/6 mice by drinking with 2.5% DSS, and the clinical symptoms such as body weight loss, diarrhea and bloody displayed. MPO activity and histologic changes in colons were detected. Compared with DSS group, bergenin (20, 50 mg/kg) and 5-ASA (100 mg/kg) significantly reduced DAI scores and MPO activity in colons of colitis mice (**Figures 1A,B**). In DSS group, the colonic length of mice was shorten, which was rescued by bergenin (20, 50 mg/kg) treatment (**Figure 1C**). Results of H&E stain analysis showed severe damage of crypts, loss of goblet cells, infiltration of mononuclear cells, and even formation of serious ulcers in the colons of colitis mice, bergenin (25, 50 mg/kg) significantly reduced the inflammation, and showed improvement trend of crypt damage (**Figure 1D**).

### Effect of Bergenin on the Expressions of Pro-inflammatory Cytokines and Barrier Function-Associated Proteins in Colons of Mice with DSS-Induced Colitis

**FIGURE 2:**
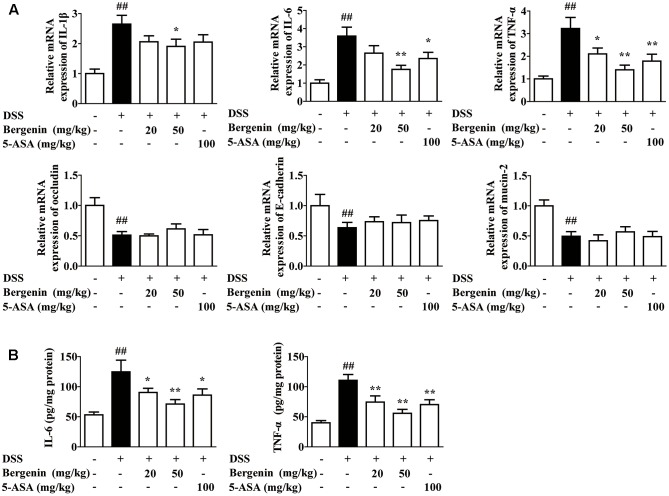
Effect of bergenin on expressions of pro-inflammatory cytokines and barrier function-associated proteins in colons of mice with DSS-induced colitis. Mice were treated with 2.5% DSS in drinking water for 7 days, provided with drinking water for another 3 days. Bergenin (20, 50 mg/kg; i.g.) and 5-ASA (100 mg/kg; i.g.) were administrated daily for consecutive 10 days. **(A)** The mRNA expressions of pro-inflammatory cytokine IL-1β, IL-6, TNF-α and barrier function-associated proteins occludin, *E*-cadherin, and MUC-2 in colons were determined by Q-PCR assay. **(B)** Protein levels of IL-6 and TNF-α were determined by ELISA. Data were presented as the means ± SEM (*n* = 6). ^##^*p* < 0.01 vs. the group without any treatment; ^∗^*p* < 0.05 and ^∗∗^*p* < 0.01 vs. DSS group.

Data indicate that persistent inflammation and damage to gastrointestinal mucosa barrier occupy important positions in the occurrence and development of UC. Therefore, we further investigated the effects of bergenin on the expressions of pro-inflammatory cytokines IL-1β, IL-6 and TNF-α and barrier function-associated proteins *E*-cadherin, occludin and MUC-2 in colons. As shown in **Figure 2A**, the mRNA expressions of IL-1β, IL-6 and TNF-α in the colons of colitis mice significantly increased, and bergenin (20, 50 mg/kg) and 5-ASA (100 mg/kg) showed obvious inhibition. The inhibitory percentage of bergenin (50 mg/kg) was 28, 51, and 57%, respectively. In contrast, bergenin only slightly affected the mRNA expressions of *E*-cadherin, occludin and MUC-2. Furthermore, the inhibitory effect of bergenin on the protein expressions of IL-6 and TNF-α in colons was confirmed by using ELISA (**Figure 2B**).

### Effect of Bergenin on the Expressions of Pro-inflammatory Cytokines in LPS-Stimulated Macrophages

**FIGURE 3:**
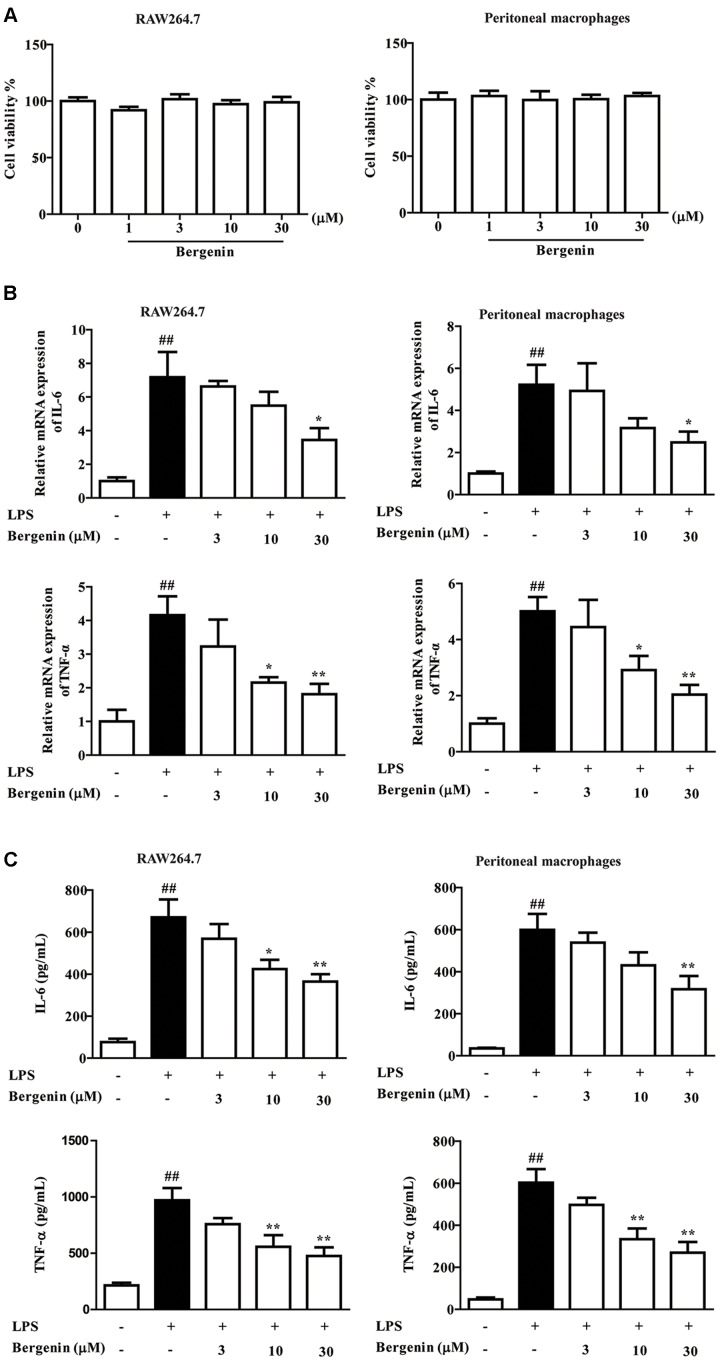
Effect of bergenin on production of LPS-induced pro-inflammatory cytokine in macrophages. **(A)** RAW264.7 cells and peritoneal macrophages isolated from mice were incubated with bergenin (1, 3, 10, 30 μM) for 24 h, and viability was determined by MTT assay. **(B)** Peritoneal macrophages and RAW264.7 cells were treated with bergenin (3, 10, 30 μM) in the presence of LPS (1 μg/mL) for 24 h. The mRNA expressions of IL-6 and TNF-α were determined by Q-PCR assay. **(C)** Protein levels of IL-6 and TNF-α in the culture medium were determined by ELISA. Data were presented as means ± SEM of three independent experiments. ^##^*p* < 0.01 vs. the group without any treatment; ^∗^*p* < 0.05 and ^∗∗^*p* < 0.01 vs. LPS group.

*In vitro* anti-inflammatory effect of bergenin was evaluated in mouse peritoneal macrophages and RAW267.4 cells, and LPS was adopted as a stimulant. At the concentrations of 1, 3, 10, 30 μM, bergenin did not affect the viability of peritoneal macrophages and RAW267.4 cells (**Figure 3A**). The protein and mRNA expressions of IL-6 and TNF-α in LPS-stimulated peritoneal macrophages and RAW267.4 cells were significantly inhibited by bergenin (10, 30 μM) treatment (**Figures 3B,C**).

### Effect of Bergenin on the Activation of NF-κB Signaling Pathway in LPS-Stimulated Macrophages

**FIGURE 4:**
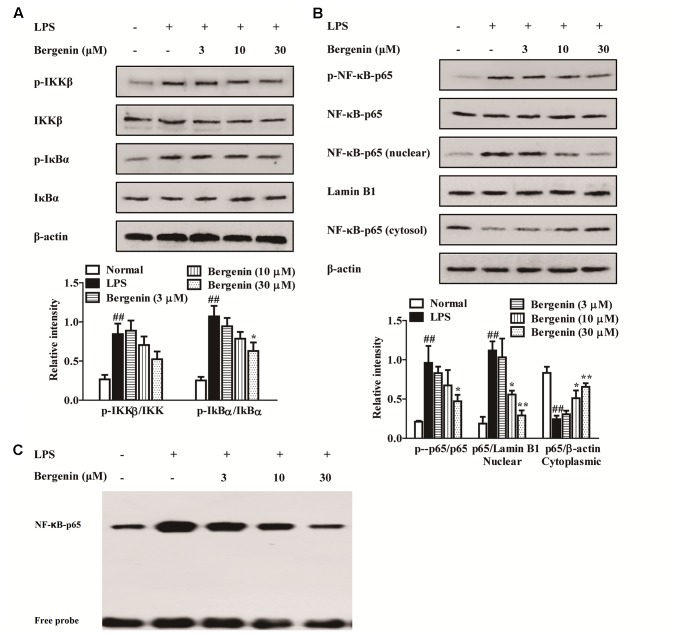
Effect of bergenin on the activation of NF-κB signaling pathway in macrophages. RAW264.7 cells were treated with bergenin (3, 10. 30 μM) for 24 h, and stimulated with LPS (1 μg/mL) for another 30 min. **(A)** Levels of phosphorylated IKKβ and IκBα were determined by western blotting assay. The β-actin was used as control. **(B)** Level of phosphorylated NF-κB-p65 and nuclear translocation of NF-κB-p65 were determined by western blotting assay. The β-actin and Lamin B1 were used as controls. **(C)** DNA-binding activity of NF-κB-p65 was determined by EMSA. Data were presented as means ± SEM of three independent experiments. ^##^*p* < 0.01 vs. the group without any treatment; ^∗^*p* < 0.05 and ^∗∗^*p* < 0.01 vs. LPS group.

The NF-κB signaling pathway plays an essential role in transcriptional induction of inflammation-related cytokines, such as IL-6 and TNF-α, and we detected the effect of bergenin on the activation of NF-κB signaling pathway. As shown in **Figure 4**, LPS stimulation induced obvious up-regulation of IKKβ, IκBα and NF-κB-p65 phosphorylations in RAW264.7 cells. Bergenin nearly did not affect the phosphorylations, but it obviously suppressed the nuclear translocation and DNA-binding activity of NF-κB-p65 at concentrations of 10 and 30 μM.

### Effects of PPARγ Antagonist GW9662 and siPPARγ on Bergenin-Inhibited Expressions of Pro-inflammatory Cytokines and Nuclear Translocation of NF-κB-p65 in LPS-Stimulated Macrophages

**FIGURE 5:**
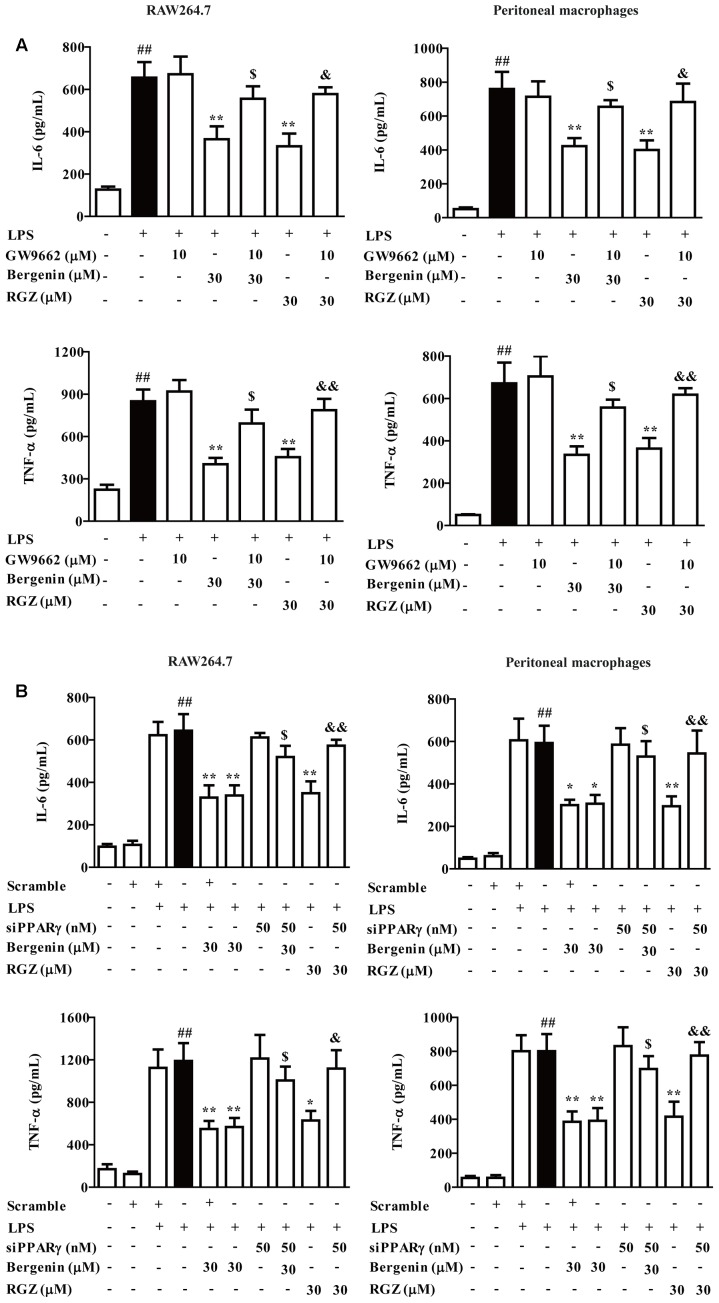
Effect of PPARγ antagonist and siPPARγ on bergenin-inhibited secretion of pro-inflammatory cytokines in macrophages. **(A)** Peritoneal macrophages and RAW264.7 cells were treated with GW9662 (10 μM) for 1 h, followed by treatment of bergenin (30 μM) for another 24 h in the presence of LPS (1 μg/mL). Protein levels of IL-6 and TNF-α in the culture medium were determined by ELISA. **(B)** Peritoneal macrophages and RAW264.7 cells were transfected with siPPARγ for 24 h, followed by treatment of bergenin (30 μM) for another 24 h in the presence of LPS (1 μg/mL). Protein levels of IL-6 and TNF-α in the culture medium were determined by ELISA. Data were presented as means ± SEM of three independent experiments. ^##^*p* < 0.01 vs. the group without any treatment; ^∗^*p* < 0.05 and ^∗∗^*p* < 0.01 vs. LPS group; ^$^*p* < 0.05 and ^$$^*p* < 0.01 vs. Bergenin group; ^&^*p* < 0.05 and ^&&^*p* < 0.01 vs. RGZ group. RGZ, rosiglitazone.

PPARγ, an important nuclear receptor, owns the power to hinder nuclear translocation and DNA-binding activity of NF-κB-p65, and subsequently down-regulate expressions of pro-inflammatory cytokines (Luo et al., 2017; Su et al., 2017). As a kind of isocoumarin, bergenin has potential to function by activating PPARγ. In this study, GW9662 (a specific PPARγ antagonist) and siPPARγ were combinately used with bergenin, and the protein expressions of IL-6 and TNF-α were determined by ELISA. Before the usage of siRNA, three pairs of siPPARγ were prepared, and transfection efficiency was detected by using Q-PCR assay. The siPPARγ-1 showed strongest down-regulation, and were selected and used in the following experiments (**Supplementary Figure S1**). As shown in **Figures 5A,B**, bergenin (30 μM) significantly reduced the protein expressions of IL-6 and TNF-α in LPS-stimulated peritoneal macrophages and RAW267.4 cells. Both GW9662 (10 M) and siPPARγ themselves had no significant effect on the expressions, but almost completely reversed the action of bergenin.

**FIGURE 6:**
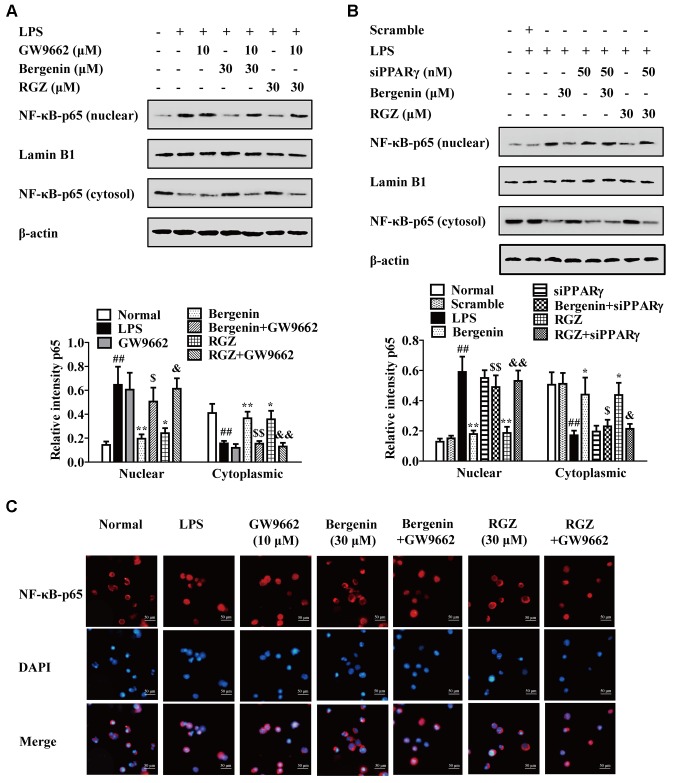
Effect of PPARγ antagonist and siPPARγ on bergenin-mediated inhibition of NF-κB-p65 nuclear translocation in macrophages. **(A)** RAW264.7 cells were incubated with GW9662 (10 μM) for 1 h, followed by treatment of bergenin (30 μM) for another 24 h, and stimulated with LPS for 30 min. Protein level of NF-κB-p65 were determined by western blotting assay. **(B)** RAW264.7 cells were transfected with siPPARγ for 24 h, followed by treatment of bergenin (30 μM) for another 24 h, and stimulated with LPS for 30 min. Protein levels of NF-κB-p65 in cytosol and nuclear were determined by western blotting assay. **(C)** RAW264.7 cells were incubated with GW9662 (10 μM) for 1 h, followed by treatment of bergenin (30 μM) for another 24 h, and stimulated with LPS for 30 min. Cells were immunostained with DAPI (blue) and NF-κB-p65 (red), and then observed by using a fluorescence microscope. Data were presented as means ± SEM of three independent experiments. ^##^*p* < 0.01 vs. the group without any treatment; ^∗^*p* < 0.05 and ^∗∗^*p* < 0.01 vs. LPS group; ^$^*p* < 0.05 and ^$$^*p* < 0.01 vs. Bergenin group; ^&^*p* < 0.05 and ^&&^*p* < 0.01 vs. RGZ group. RGZ, rosiglitazone.

Subsequently, we confirmed the involvement of PPARγ in action of bergenin. As shown in **Figures 6A,B**, both GW9662 and siPPARγ significantly diminished the inhibitory effects of bergenin on the nuclear translocation of NF-κB-p65 in LPS-stimulated RAW264.7 cells, which was supported by an immunofluorescence assay (**Figure 6C**).

### Activation of Bergenin on PPARγ

**FIGURE 7:**
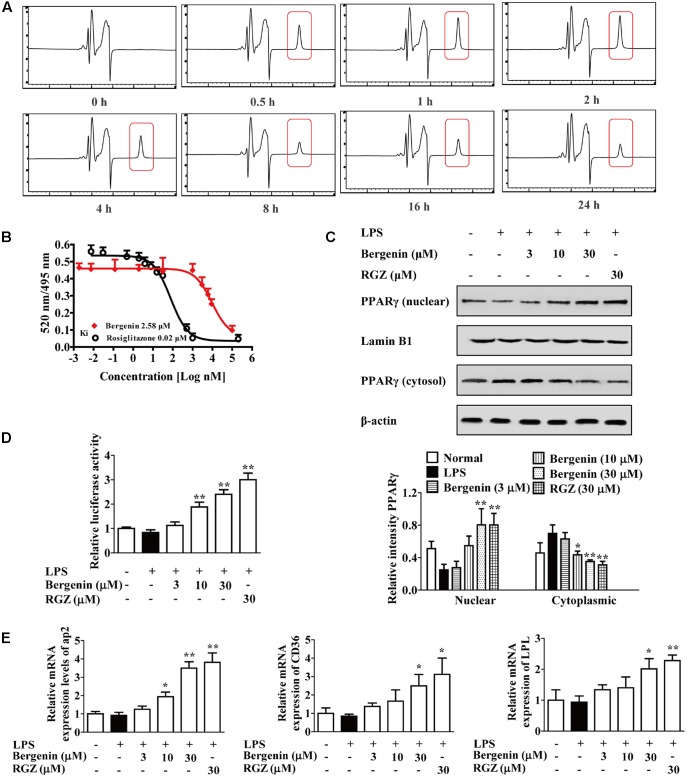
Activation of PPARγ by bergenin in macrophages. **(A)** RAW264.7 cells were treated with bergenin (30 μM) for 0, 0.5, 1, 2, 4, 8, 16, and 24 h, and harvested and lysed. The lysates were subjected to HPLC analysis for determining endocytosis of bergenin. **(B)** Competitive binding assay for PPARγ. **(C–E)** RAW264.7 cells were incubated with bergenin (3, 10, 30 μM) or rosiglitazone (30 μM) for 24 h in the present or absent of LPS (1 μg/mL). Protein levels of PPARγ in cytosol and nucleus were determined by western blotting assay **(C)**; Transcriptional activity of PPARγ was determined by luciferase assay **(D)**; The mRNA expressions of CD36, LPL and ap2 were determined by Q-PCR assay **(E)**. Data were presented as means ± SEM of three independent experiments. ^##^*p* < 0.01 vs. the group without any treatment; ^∗^*p* < 0.05 and ^∗∗^*p* < 0.01 vs. LPS group. RGZ, rosiglitazone.

Then, we explored the possibility that bergenin directly activate PPAR-γ in macrophages. PPAR-γ mainly located at the cytoplasm, and the endocytosis of bergenin was firstly detected. RAW264.7 cells were treated with bergenin (30 μM) for 0, 0.5, 1, 2, 4, 8, 16, and 24 h, washed with PBS for three times to remove bergenin or others that attached to the membrane, and disrupted by multigelation for five times to lysate cells and obtain the intracellular substances. Then, they were centrifuged to further remove the cell membranes, supernatant was collected, and HPLC assay was performed. As shown in **Figure 7A**, the obvious peaks of bergenin appeared at 0.5–24 h, and the maximum peak appeared at about 1–2 h. All these implied that bergenin could enter into the inside of macrophages and met the necessary requirement for activating PPAR-γ.

Next, the binding to PPARγ, and the effects of bergenin on the nuclear translocation and transcriptional activity of PPARγ and the expressions of CD36, LPL and aP2 were investigated. As shown in **Figure 7B**, the results of competitive ligand-binding assay showed that bergenin competed with rosiglitazone for binding to PPAR-γ (rosiglitazone was used as positive control with a Ki value of 0.02 μM; bergenin displaced rosiglitazone from the PPAR-γ LBD with a Ki value of 2.58 μM). In addition, as illustrated in **Figures 7C–E**, bergenin obviously promoted nuclear translocation and transcriptional activity of PPARγ, increased mRNA expressions of aP2, CD36 and LPL in a concentration-dependent manner. Taken together, all these results suggested that bergenin might be a PPARγ agonist.

### Mechanisms by Which Bergenin Inhibited Nuclear Translocation of NF-κB-p65 after Activating PPARγ

Recently, a series of post-translational modifications such as acetylation, methylation phosphorylation and ubiquitination have been reported. Among them, acetylation is important for the modification of NF-κB in inflammatory response, and might be regulated by PPARγ. Data indicates that inhibition of NF-κB-p65 acetylation could improve the association of NF-κB-p65 and IκBα and down-regulate the nuclear translocation of NF-κB-p65.

**FIGURE 8:**
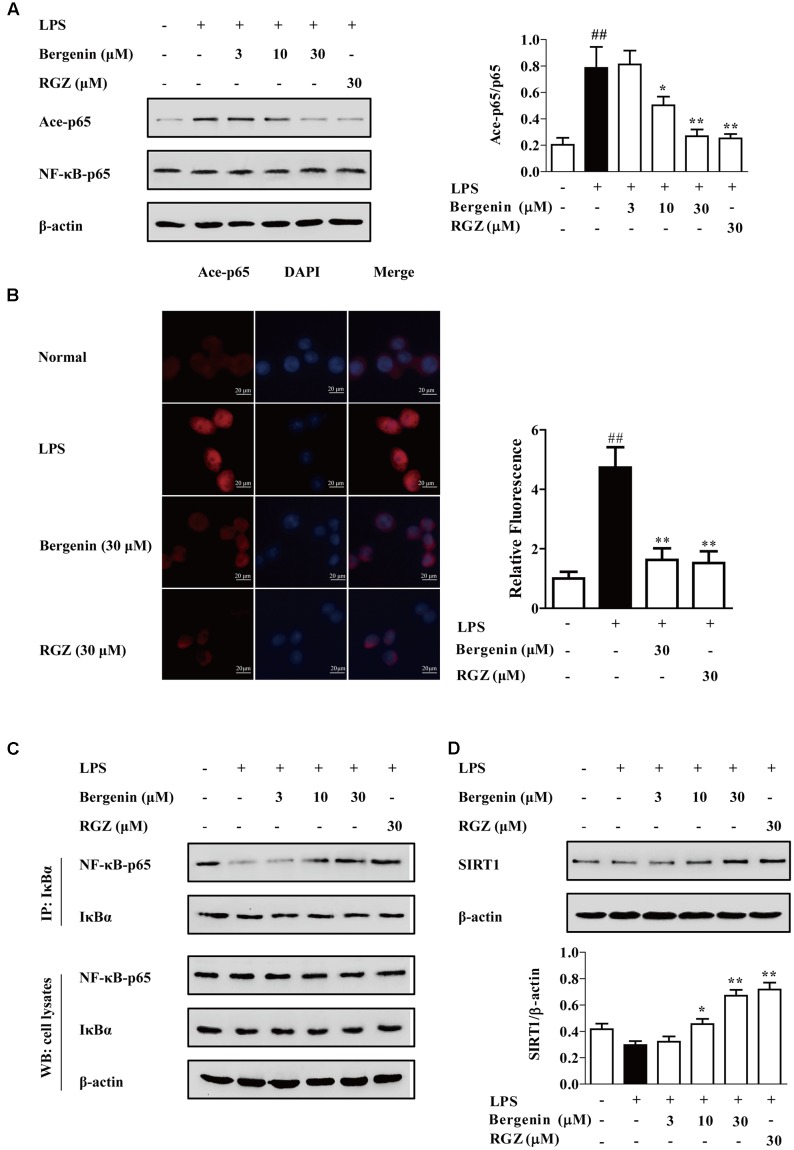
Effect of bergenin on level of Ace-p65, association of NF-κB-p65 and IκBα, and level of SIRT1 in LPS-stimulated macrophages. RAW264.7 cells were incubated with bergenin (1, 3, 10, and 30 μM) or rosiglitazone (30 μM) for 24 h, and stimulated with LPS for 30 min. **(A,B)** Level of Ace-p65 was determined by western blotting and fluorescence microscope assay. **(C)** Association of NF-κB-p65 and IκBα was detected by co-immunoprecipitation assay. **(D)** Level of SIRT1 was determined by western blotting assay. ^##^*p* < 0.01 vs. the group without any treatment; ^∗^*p* < 0.05 and ^∗∗^*p* < 0.01 vs. LPS group.

To deeply reveal the mechanisms by which bergenin hindered the nuclear translocation of NF-κB-p65 after activating PPARγ and subsequent inhibited the expressions of IL-6 and TNF-α, the following experiments were performed. The results of western blotting and immunofluorescence assays showed that bergenin (10, 30 μM) significantly inhibited the acetylation of NF-κB-p65 in LPS-stimulated RAW264.7 cells, which was accompanied by improvement of NF-κB-p65 and IκBα association (**Figures 8A–C**).

**FIGURE 9:**
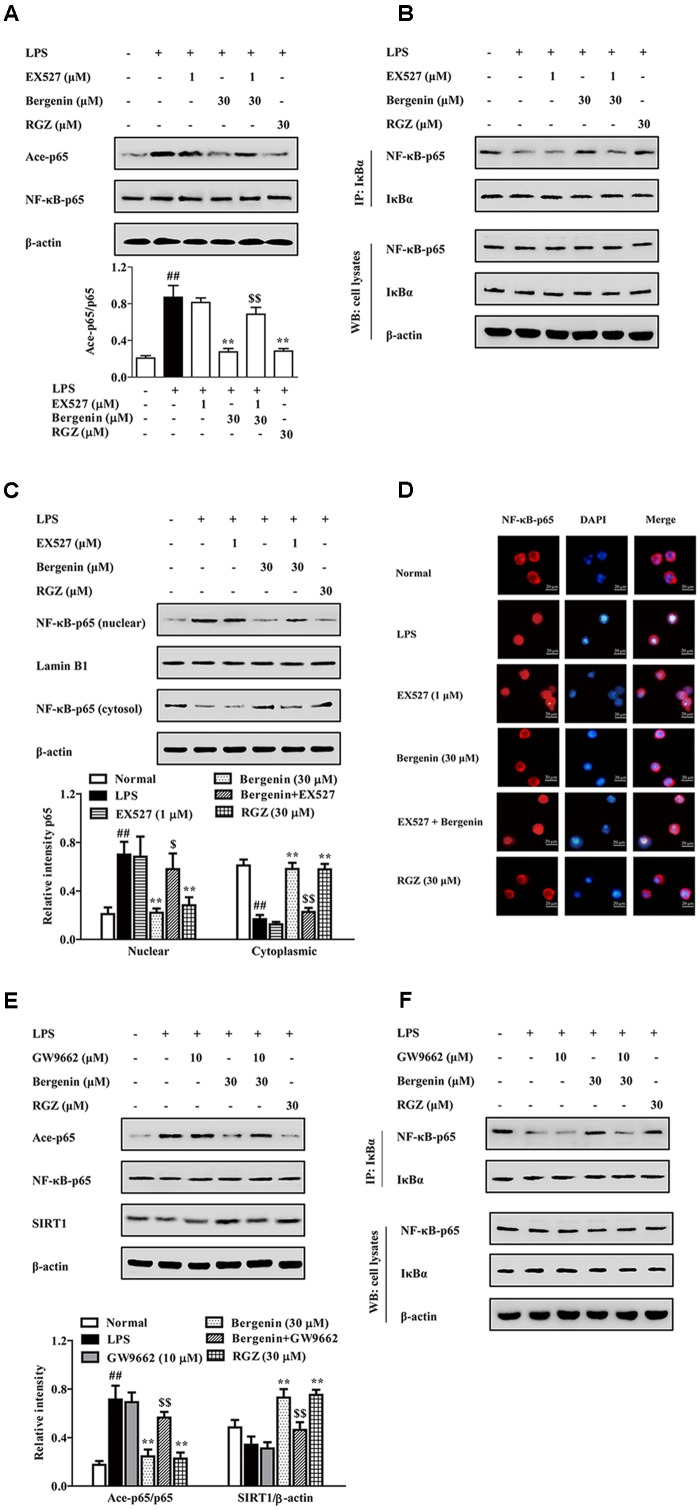
The participation of SIRT1 and PPARγ in bergenin-inhibited nuclear translocation of NF-κB-p65 after activating PPARγ in macrophages. **(A–D)** RAW264.7 cells were incubated with EX527 (1 μM) for 1 h, followed by treatment of bergenin (30 μM) for another 24 h, and stimulated with LPS for 30 min. Ace-p65 level and association of NF-κB-p65 and IκBα were determined by western blotting and co-immunoprecipitation assays **(A,B)**; Nuclear translocation of NF-κB-p65 was observed by western blotting and immunofluorescence assays **(C,D)**. **(E,F)** RAW264.7 cells were incubated with GW9662 (10 μM) for 1 h, and followed by treatment of bergenin (30 μM) for another 24 h, and stimulated with LPS for 30 min. Levels of Ace-p65 and SIRT1 were determined by western blotting assay **(E)**; Association of NF-κB-p65 and IκBα was determined by co-immunoprecipitation assay **(F)**. Data were presented as means ± SEM of three independent experiments. ^##^*p* < 0.01 vs. the group without any treatment; ^∗^*p* < 0.05 and ^∗∗^*p* < 0.01 vs. LPS group; ^$^*p* < 0.05 and ^$$^*p* < 0.01 vs. Bergenin group. RGZ, rosiglitazone.

SIRT1, a NAD^+^-dependent class III protein deacetylase, regulates multiple biological effects by modulating histones and non-histone substrates. PPARγ agonists rosiglitazone and pioglitazone could increase SIRT1 expressions in multiple tissues and cells (Ghizzoni et al., 2011; Huang et al., 2012). As shown in **Figure 8D**, bergenin (10, 30 μM) significantly increased the expression of SIRT1 in LPS-stimulated RAW264.7 cells. Next, EX527, a specific SIRT1 inhibitor, was used. As shown in **Figures 9A–D**, EX527 (1 μM) markedly diminished bergenin-induced inhibition of acetylation and nuclear translocation of NF-κB-p65, and the up-regulation of the association between NF-κB-p65 and IκBα.

Then, we confirmed that the above-mentioned action of bergenin was PPARγ-dependent by using GW9662. The results showed that GW9662 obviously weakened bergenin-mediated inhibition of acetylation and nuclear translocation of NF-κB-p65, and up-regulation of SIRT1 expression as well as association of NF-κB-p65 and IκBα in LPS-stimulated RAW264.7 cells (**Figures 9E,F**).

Taken together, after activating PPARγ, bergenin up-regulated the expression of SIRT1, inhibited the acetylation of NF-κB-p65, increased the association of NF-κB-p65 and IκBα, and hindered the nuclear translocation of NF-κB-p65 in LPS-stimulated RAW264.7 cells.

### Effects of PPARγ Antagonist GW9662 on Bergenin-Mediated Inhibition of DSS-Induced Colitis in Mice

**FIGURE 10:**
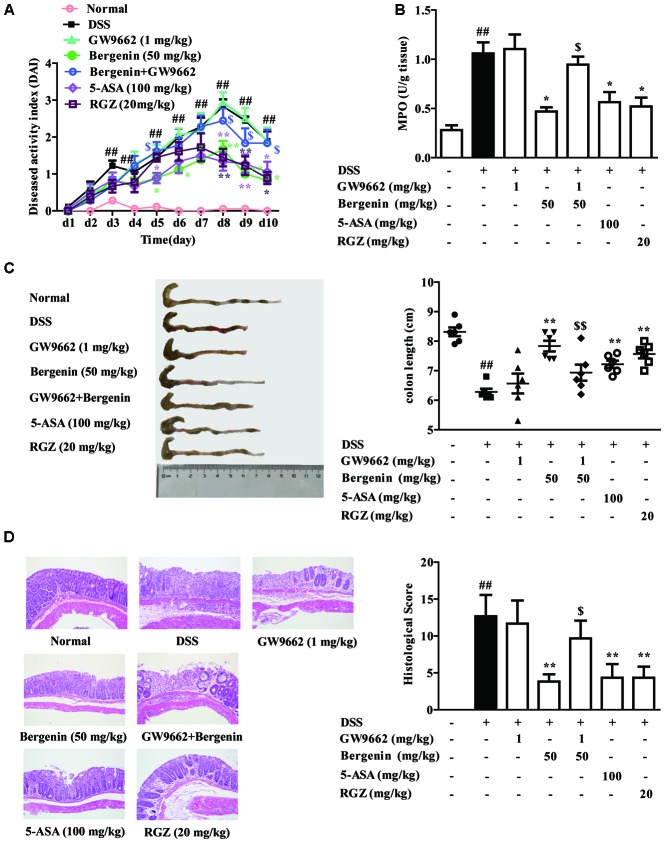
Effect of GW9662 on bergenin-mediated inhibition of mice with DSS-induced colitis. **(A–C)** DAI scores, MPO activity and the length of colons. **(D)** Representative images were showed pathologic abnormalities with H&E staining in colons. Histological score were analyzed from H&E staining. Data were presented as the means ± SEM (*n* = 6). ^##^*p* < 0.01 vs. the group without any treatment; ^∗^*p* < 0.05 and ^∗∗^*p* < 0.01 vs. DSS group; ^$^*p* < 0.05 and ^$$^*p* < 0.01 vs. Bergenin group. RGZ, rosiglitazone.

Finally, to confirm the key role that PPARγ played in bergenin-mediated protection of colitis, the model of DSS-induced colitis was established in mice, bergenin was combined with GW9662, and 5-ASA and rosiglitazone were taken as positive drugs. Oral administration of bergenin (50 mg/kg), 5-ASA (100 mg/kg) and rosiglitazone (20 mg/kg) significantly improved disease symptoms of mice with colitis, including reducing DAI score and MPO activity, rescuing the shortening of colon length, and alleviating histopathological changes in colons. GW9662 (1 mg/kg) itself had no significant effect, but dramatically weakened the action of bergenin (**Figures 10A–D**).

**FIGURE 11:**
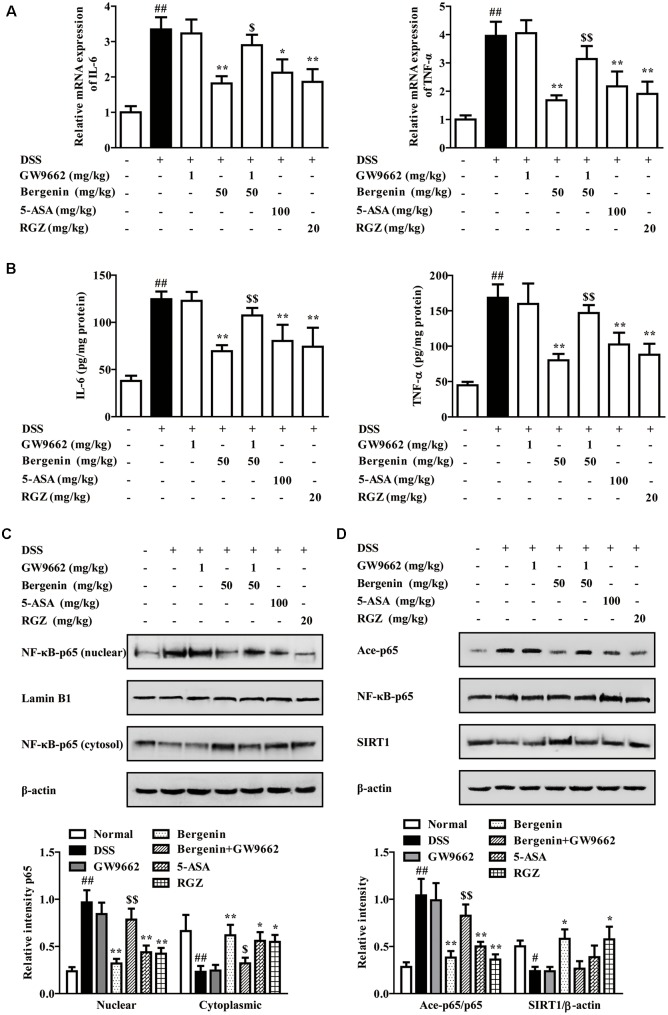
Effect of GW9662 on bergenin-regulated production of pro-inflammatory cytokines and activation of related signals in colons of mice with DSS-induced colitis. **(A)** The mRNA expressions of IL-6 and TNF-α in colons of colitis mice were determined by Q-PCR assay. **(B)** Protein levels of IL-6 and TNF-α in colons of colitis mice were determined by ELISA. **(C)** Nuclear translocation of NF-κB-p65 in colons of colitis mice was determined by western blotting assay. **(D)** Levels of SIRT1 and Ace-p65 in colons of colitis mice were determined by western blotting assay. Data were presented as the means ± SEM (*n* = 6). ^##^*p* < 0.01 vs. the group without any treatment; ^∗^*p* < 0.05 and ^∗∗^*p* < 0.01 vs. DSS group; ^$^*p* < 0.05 and ^$$^*p* < 0.01 vs. Bergenin group. RGZ, rosiglitazone.

**FIGURE 12:**
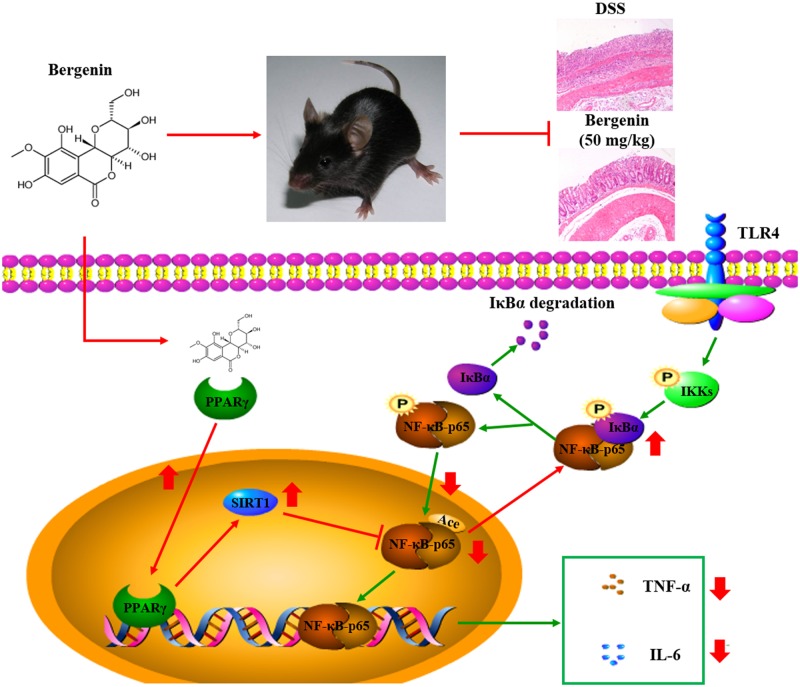
Mechanisms for the anti-ulcerative colitis (UC) effect of bergenin. Bergenin activates PPARγ leading to increased expression of SIRT1. SIRT1 reduces the acetylation of NF-κB-65, up-regulates the association of NF-κB-p65 and IKBα, and prevents the nuclear translocation of NF-κB-p65. This leads to reduced expression of TNF-α and IL-6, and the subsequent anti-colitis action of bergenin

As shown in **Figures 11A,B**, bergenin (50 mg/kg), 5-ASA (100 mg/kg) and rosiglitazone (20 mg/kg) significantly down-regulated the mRNA and protein expressions of IL-6 and TNF-α in colons of mice with DSS-induced colitis. When combined with GW9662 (1 mg/kg), the action of bergenin disappeared. In addition, GW9662 (1 mg/kg) obviously reversed bergenin-mediated inhibition of NF-κB-p65 acetylation and nuclear translocation, up-regulation of SIRT1 expression in colon tissues of colitis mice (**Figures 11C,D**). The main mechanisms about anti-UC effect of bergenin has been showed in **Figure 12**.

## Discussion

Recently, the incidence of UC rises year by year with the changes of dietary structure and environment. In clinic, 5-aminosalicylic acid (5-ASA), corticosteroids, immunosuppressive agents and biological agents are usually used for UC treatment. However, many problems have arisen, especially in terms of cost and patients’ increased susceptibility to infection as well as occurrence of low-responders. New therapeutic agents with high efficiency and few side effects are expected (Galli et al., 2011; Roberts-Thomson et al., 2011). In the progress of drug discovery, the model of DSS-induced UC in mice is usually adopted, which is established easily with high rate of success and similarity to the characteristics of human UC. The disease symptoms of mice rapidly emergence in day 2 after the DSS administration, and are in conformity with the characteristics of acute colitis. The time interval of drug administration was from day 1 to 10. Bergenin is the major bioactive ingredient in bergenia purpurascens. In the present study, bergenin showed well alleviation of colitis induced by DSS in mice, evidenced by reduced DAI scores, shortening of colon length, MPO activity and pathologic abnormalities in colons. In UC, the characteristic of mucosal or submucosal sites in colons mainly include diffuse inflammation, reduction of goblet cells and crypt structure damage. The results of H&E examination revealed that bergenin could significantly reduce the infiltration of inflammatory cells, and only showed improvement trend of crypt damage. In addition, bergenin only inhibited the DSS-induced expression of pro-inflammatory cytokines IL-6 and TNF-α in colons, but did not influence the reduced expression of barrier function-associated proteins occludin, E-cadherin and MUC-2. All these results implied that bergenin could attenuate UC in mice mainly by inhibition of inflammatory response.

IL-6 and TNF-α, mainly secreted by macrophages (MQs), are key pro-inflammatory cytokines responsible for the inflammation of colonic mucosa and destruction of mucosal barrier. In UC patients, MQs are over-activated under the stimulation of bacterial endotoxin in colonic lamina propria and mucosa. In normal state, MQs show less response to inflammatory signals and promote the generation of immunosuppressive cells. However, in inflammatory state, the function of MQs changes, produce multiple pro-inflammatory cytokines including IL-6 and TNF-α, and participate in the development of inflammation in UC and other inflammation-related diseases (Rivollier et al., 2012; Zhang et al., 2016). IL-6 activates the STAT-3 pathway to induce the expression of intercellular adhesion molecules ICAM-1 and strengthen the neutrophil adhesion; It also reduces the secretion of electrolytes in epithelial cells, increases the permeability of membrane, and leads to damage of tissue (Hwang et al., 2014). In addition, TNF-α can promote the accumulation of neutrophils at the inflammatory site, decomposition of junction proteins, consequent destruction of epithelial barrier (Uwada et al., 2017). Then, effect of bergenin on LPS-stimulated RAW264.7 cells and peritoneal macrophages was detected. However, pharmacokinetic data of bergenin in mice are not reported, and the plasma concentrations obtained with the highest dose of bergenin administered (50 mg/kg) are thus not known. Therefore, concentrations of bergenin used *in vitro* experiments were according to references (Jain et al., 2014; Yang et al., 2017), and 3, 10, 30 μM were selected. Results showed that bergenin (10, 30 μM) obviously inhibited the expressions of IL-6 and TNF-α in LPS-stimulated peritoneal macrophages and RAW267.4 cells.

Under the condition of LPS stimulation, multiple inflammation-related signals are activated. Among them, NF-κB signaling pathway occupies a key position. In resting cells, NF-κB p50/p65 heterodimers, present in the cytoplasm, are in an inactive state by binding to inhibitory proteins IκBs. Diverse external stimuli can induce the phosphorylation of IκB kinase (IKK), and increase its enzyme activity. Then, IKK phosphorylates IκB protein, leading to their subsequent ubiquitination and degradation, and release of the NF-κB-p50/p65 dimer. Subsequently, NF-κB-p65 translocates into the nucleus, binding to the DNA binding site of target genes including IL-6 and TNF-α (Dubuquoy et al., 2003). In addition, NF-κB pathway is over-activated in UC patients and animal models, and therapeutic approach targeting at inhibiting activation of NF-κB signaling pathway will be effective for UC therapy (Qian et al., 2016). In this study, bergenin inhibited the phosphorylation of IKKβ and IκBα, and the phosphorylation, nuclear translocation and DNA binding activity of NF-κB-p65. Furthermore, bergenin-mediated inhibition of nuclear translocation and DNA binding activity of NF-κB-p65 was stronger.

PPARγ is an important member of the nuclear receptor family, and acts in ligand-dependence. In resting state, PPARγ mainly distributes in the cytoplasm. After binding with ligand, it translocates from cytoplasm into nucleus, binds as a heterodimer with the retinoid X receptor to specific DNA response elements (PPREs) within promoters of target genes such as CD36 and aP2. In UC patients and experimental colitis models, the expression of PPARγ showed impaired features in colons; specific knockout of PPARγ in macrophages and epithelial cells of UC mice significantly aggravated the disease symptoms and pathological damages (Dubuquoy et al., 2006). Furthermore, activation of PPARγ could significantly reduce LPS-induced expressions of IL-6 and TNF-α in macrophages (Hu et al., 2013); PPARγ bound to its transcriptional element, activated the polarization gene of M2 type, promoted the transformation of M1 to M2 type, and inhibited inflammation (Bouhlel et al., 2007). All these characteristics of PPARγ have been revealed in PPARγ agonists rosiglitazone and pioglitazone (Adachi et al., 2006; Lewis et al., 2008). Bergenin is an isocoumarin, and shows the characteristic of polyphenols at the chemical structure. Wang et al. (2015) reported that sparstolonin B, an isocoumarin, significantly inhibited LPS-induced expressions of MCP-1, IL-6 and TNF-α in 3T3-L1 adipocytes by activating PPARγ; Liu et al. (2014) reported that curcumin inhibited the expressions of inflammatory mediators and neuron apoptosis by increasing the nuclear translocation and the transcriptional activity of PPARγ; Serra et al. (2016) reported that resveratrol activated PPARγ to up-regulate the ratio of GSH/GSSG and decrease the level of ROS. Based on this, we explored the key role of PPARγ in bergenin-mediated anti-inflammatory effects. The results showed that GW9662 and siPPARγ significantly weakened bergenin-mediated inhibition of IL-6 and TNF-α expressions, and NF-κB-p65 nuclear translocation in LPS-stimulated peritoneal macrophages and RAW264.7 cells. Furthermore, bergenin was demonstrated to be a PPARγ agonist because it could enter into the cytoplasm, directly bind with PPARγ, promote nuclear translocation and transcriptional activity of PPARγ, and up-regulate expressions of target gene aP2, CD36 and LPL.

Up to now, the inhibition of NF-κB-p65 nuclear translocation is attributed to reduction of IκBα degradation or regulation of post-translational modification of NF-κB-p65 (Lu et al., 2013; Lakshmi et al., 2014; Zhang et al., 2016). Our above-mentioned results indicated that bergenin only showed slight effect on IκBα degradation. Therefore, we investigated the effect of bergenin on post-translational modification of NF-κB-p65 in LPS-stimulated RAW264.7 cells. Histone deacetylases (HDACs) are an important member of a group of enzymes that modify chromatin conformation, HDAC3 could inhibit the acetylation of NF-κB-p65 in TNF-α-induced COS-7 cells. SIRT1, a member of HDAC3, exerts effects depending on nicotinine adenine dinucleotide (NAD^+^). When SIRT1 is at high level, Ace-p65 significantly decreases in nucleus, association of NF-κB-p65 and IκBα increases, and NF-κB-p65 translocates to cytoplasm (Chen et al., 2001; Lu and Lin, 2010). Under condition of HDAC3 high expression, knockout of IκBα could prevent the nuclear translocation of NF-κB-p65. In addition, SIRT1 inhibits the production of inflammatory mediators, and pioglitazone and rosiglitazone could promote the expression of SIRT1 in multiple tissues and cells (Huang et al., 2012; Zhang et al., 2016). Our current results showed that bergenin (10, 30 μM) significantly decreased the level of acetylated NF-κB-p65 and increased the level of SIRT1 in LPS-induced RAW264.7 cells, and EX527 almost reversed the bergenin-mediated reduction of acetylated NF-κB-p65 level and nuclear translocation of NF-κB-p65. In addition, in combination with GW9662, the regulation of bergenin on the levels of acetylated NF-κB-p65 and SIRT1 almost disappeared. Finally, DSS-induced colitis was established in mice, bergenin was orally administered in combination with GW9662 to explicit the participation of above signal molecules in the anti-colitis effect of bergenin. The results showed that GW9662 itself did not affect the disease symptoms of mice with colitis, but significantly weakened bergenin-mediated regulation of levels of IL-6, TNF-α, acetylated NF-κB-p65, SIRT1 and nuclear translocation of NF-κB-p65 in colons, and consequent anti-colitis effect.

## Conclusion

In summary, bergenin can significantly attenuate DSS-induced colitis in mice. The mechanism might be as follows: activation of PPARγ, up-regulation of SIRT1 expression, inhibition of NF-κB-p65 acetylation and nuclear translocation, thereby reduction of the expression of pro-inflammatory cytokines IL-6 and TNF-α.

## Author Contributions

Z-fW and YD designed the study. KW and Y-fL performed the experiments and wrote the manuscript. QL and X-mL analyzed the data.

## Conflict of Interest Statement

The authors declare that the research was conducted in the absence of any commercial or financial relationships that could be construed as a potential conflict of interest.

## Supplementary Material

FIGURE S1 Effect of siPPAR-γ 1–3 on mRNA expression of PPAR-γ in **(A)** RAW264.7 cells and **(B)** peritoneal macrophages. Peritoneal macrophages and RAW264.7 cells were transfected with siPPARγ 1–3 for 24 h, and mRNA expression of PPAR-γ was detected by using Q-PCR assay. ^∗^*p* < 0.05 and ^∗∗^*p* < 0.01 vs. the group without any treatment.

## References

[B1] Adachi M. Kurotani R. Morimura K. Shah Y. Sanford M. Madison B. B. (2006). Peroxisome proliferator activated receptor gamma in colonic epithelial cells protects against experimental inflammatory bowel disease. *Gut* 55 1104–1113. 10.1136/gut.2005.081745 16547072 PMC1513267

[B2] Ananthakrishnan A. N. (2015). Epidemiology and risk factors for IBD. *Nat. Rev. Gastroenterol. Hepatol.* 12 205–217. 10.1038/nrgastro.2015.34 25732745

[B3] Baumgart D. C. Sandborn W. J. (2007). Inflammatory bowel disease: clinical aspects and established and evolving therapies. *Lancet* 369 1641–1657. 10.1016/S0140-6736(07)60751-X 17499606

[B4] Bertin B. Dubuquoy L. Colombel J. F. Desreumaux P. (2013). PPAR-gamma in ulcerative colitis: a novel target for intervention. *Curr. Drug Targets* 14 1501–1517. 10.2174/13894501113149990162 23651165

[B5] Bonfield T. L. Thomassen M. J. Farver C. F. Abraham S. Koloze M. T. Zhang X. (2008). Peroxisome proliferator-activated receptor-gamma regulates the expression of alveolar macrophage macrophage colony-stimulating factor. *J. Immunol.* 181 235–242. 10.4049/jimmunol.181.1.235 18566389 PMC2819287

[B6] Bouhlel M. A. Derudas B. Rigamonti E. Dièvart R. Brozek J. Haulon S. (2007). PPARgamma activation primes human monocytes into alternative M2 macrophages with anti-inflammatory properties. *Cell Metab.* 6 137–143. 10.1016/j.cmet.2007.06.010 17681149

[B7] Chen L. F. Fischle W. Verdin E. Greene W. C. (2001). Duration of nuclear NF-kappaB action regulated by reversible acetylation. *Science* 293 1653–1657. 10.1126/science.1062374 11533489

[B8] Dubuquoy L. Jansson E. A. Deeb S. Rakotobe S. Karoui M. Colombel J. F. (2003). Impaired expression of peroxisome proliferator-activated receptor gamma in ulcerative colitis. *Gastroenterology* 124 1265–1276. 10.1016/S0016-5085(03)00271-3 12730867

[B9] Dubuquoy L. Rousseaux C. Thuru X. Peyrin-Biroulet L. Romano O. Chavatte P. (2006). PPARgamma as a new therapeutic target in inflammatory bowel diseases. *Gut* 55 1341–1349. 10.1136/gut.2006.093484 16905700 PMC1860011

[B10] Galli S. J. Borregaard N. Wynn T. A. (2011). Phenotypic and functional plasticity of cells of innate immunity: macrophages, mast cells and neutrophils. *Nat. Immunol.* 12 1035–1044. 10.1038/ni.2109 22012443 PMC3412172

[B11] Gao X. J. Guo M. Y. Zhang Z. C. Wang T. C. Cao Y. G. Zhang N. S. (2015). Bergenin plays an anti-inflammatory role via the modulation of MAPK and NF-κB signaling pathways in a mouse model of LPS-induced mastitis. *Inflammation* 38 1142–1150. 10.1007/s10753-014-0079-8 25487780

[B12] Ghizzoni M. Haisma H. J. Maarsingh H. Dekker F. J. (2011). Histone acetyltransferases are crucial regulators in NF-κB mediated inflammation. *Drug Discov. Today* 16 504–511. 10.1016/j.drudis.2011.03.009 21477662 PMC5218544

[B13] Groeger D. O’Mahony L. Murphy E. F. Bourke J. F. Dinan T. G. Kiely B. (2013). Bifidobacterium infantis 35624 modulates host inflammatory processes beyond the gut. *Gut Microbes* 4 325–339. 10.4161/gmic.25487 23842110 PMC3744517

[B14] Hu K. Yang Y. Tu Q. Luo Y. Ma R. (2013). Alpinetin inhibits LPS-induced inflammatory mediator response by activating PPAR-γ in THP-1-derived macrophages. *Eur. J. Pharmacol.* 721 96–102. 10.1016/j.ejphar.2013.09.049 24104193

[B15] Huang W. Shang W. L. Wang H. D. Wu W. W. Hou S. X. (2012). SIRT1 overexpression protects murine osteoblasts against TNF-α-induced injury *in vitro* by suppressing the NF-κB signaling pathway. *Acta Pharmacol. Sin.* 33 668–674. 10.1038/aps.2011.189 22447223 PMC4010359

[B16] Hwang J. S. Lee W. J. Kang E. S. Ham S. A. Yoo T. Paek K. S. (2014). Ligand-activated peroxisome proliferator-activated receptor-*δ* and -*γ* inhibit lipopolysaccharide-prime release of high mobility group box 1 through upregulation of SIRT1. *Cell Death Dis.* 2:e1432. 10.1038/cddis.2014.406 25275593 PMC4649513

[B17] Jain S. K. Singh S. Khajuria A. Guru S. K. Joshi P. Meena S. (2014). Pyrano-isochromanones as IL-6 inhibitors: synthesis, in vitro and in vivo antiarthritic activity. *J. Med. Chem.* 57 7085–7097. 10.1021/jm500901e 25111439

[B18] Komaki Y. Yamada A. Komaki F. Micic D. Ido A. Sakuraba A. (2017). Systematic review with meta-analysis: the efficacy and safety of CT-P13, a biosimilar of anti-tumour necrosis factor-α agent (infliximab), in inflammatory bowel diseases. *Aliment. Pharmacol. Ther.* 45 1043–1057. 10.1111/apt.13990 28239873

[B19] Lakshmi S. P. Reddy A. T. Zhang Y. Sciurba F. C. Mallampalli R. K. Duncan S. R. (2014). Down-regulated peroxisome proliferator-activated receptor γ (PPARγ) in lung epithelial cells promotes a PPARγ agonist-reversible proinflammatory phenotype in chronic obstructive pulmonary disease (COPD). *J. Biol. Chem.* 289 6383–6393. 10.1074/jbc.M113.536805 24368768 PMC3945305

[B20] Lee Y. J. Ko E. H. Kim J. E. Kim E. Lee H. Choi H. (2012). Nuclear receptor PPARγ regulated monoacylglycerol *O*-acyltransferase1 (MGAT1) expression is responsible for the lipid accumulation in diet-induced hepatic steatosis. *Proc. Natl. Acad. Sci. U.S.A.* 109 13656–13661. 10.1073/pnas.1203218109 22869740 PMC3427113

[B21] Lewis J. D. Lichtenstein G. R. Deren J. J. Sands B. E. Hanauer S. B. Katz J. A. (2008). Rosiglitazone for ulcerative colitis study group. Rosiglitazone for active ulcerative colitis: a randomized placebo-controlled trial. *Gastroenterology* 34 688–695. 10.1053/j.gastro.2007.12.012 18325386 PMC2276587

[B22] Liu Z. J. Liu H. Q. Xiao C. Fan H. Z. Huang Q. Liu Y. H. (2014). Curcumin protects neurons against oxygen-glucose deprivation/reoxygenation-induced injury through activation of peroxisome proliferator-activated receptor-γ function. *J. Neurosci. Res.* 92 1549–1559. 10.1002/jnr.23438 24975470

[B23] Lu S. P. Lin S. J. (2010). Regulation of yeast sirtuins by NAD^+^ metabolism and calorie restriction. *Biochim. Biophys. Acta* 1804 1567–1575. 10.1016/j.bbapap.2009.09.030 19818879 PMC2886167

[B24] Lu Y. Zhou Q. Zhong F. Guo S. Hao X. Li C. (2013). 15-Deoxy-Δ^12,14^-prostaglandin J_2_ modulates lipopolysaccharide-induced chemokine expression by blocking nuclear factor-κB activation via peroxisome proliferator activated receptor-γ-independent mechanism in renal tubular epithelial cells. *Nephron Exp. Nephrol.* 123 1–10. 10.1159/000353232 23887394

[B25] Luo W. Xu Q. Wang Q. Wu H. Hua J. (2017). Effect of modulation of PPAR-γ activity on Kupffer cells M1/M2 polarization in the development of non-alcoholic fatty liver disease. *Sci. Rep.* 7:44612. 10.1038/srep44612 28300213 PMC5353732

[B26] Nazir N. Koul S. Qurishi M. A. Taneja S. C. Ahmad S. F. Bani S. (2007). Immunomodulatory effect of bergenin and norbergenin against adjuvant-induced arthritis–a flow cytometric study. *J. Ethnopharmacol.* 112 401–405. 10.1016/j.jep.2007.02.023 17408893

[B27] Nevin D. K. Peters M. B. Carta G. Fayne D. Lloyd D. G. (2012). Integrated virtual screening for the identification of novel and selective peroxisome proliferator-activated receptor (PPAR) scaffolds. *J. Med. Chem.* 55 4978–4989. 10.1021/jm300068n 22582973

[B28] Nishimoto N. Nakahara H. Yoshio-Hoshino N. Mima T. (2008). Successful treatment of a patient with takayasu arteritis using a humanized anti-interleukin-6 receptor antibody. *Arthritis Rheum.* 58 1197–1200. 10.1002/art.23373 18383395

[B29] Pedersen G. Brynskov J. (2010). Topical rosiglitazone treatment improves ulcerative colitis by restoring peroxisome proliferator-activated receptor-gamma activity. *Am. J. Gastroenterol.* 105 1595–1603. 10.1038/ajg.2009.749 20087330

[B30] Qian J. Zhao W. Miao X. Li L. Zhang D. (2016). Sam68 modulates apoptosis of intestinal epithelial cells via mediating NF-κB activation in ulcerative colitis. *Mol. Immunol.* 75 48–59. 10.1016/j.molimm.2016.05.011 27235792

[B31] Rivollier A. He J. Kole A. Valatas V. Kelsall B. L. (2012). Inflammation switches the differentiation program of Ly6Chi monocytes from antiinflammatory macrophages to inflammatory dendritic cells in the colon. *J. Exp. Med.* 209 139–155. 10.1084/jem.20101387 22231304 PMC3260867

[B32] Roberts-Thomson I. C. Fon J. Uylaki W. Cummins A. G. Barry S. (2011). Cells, cytokines and inflammatory bowel disease: a clinical perspective. *Expert Rev. Gastroenterol. Hepatol.* 5 703–716. 10.1586/egh.11.74 22017698

[B33] Rousseaux C. Lefebvre B. Dubuquoy L. Lefebvre P. Romano O. Auwerx J. (2005). Intestinal antiinflammatory effect of 5-aminosalicylic acid is dependent on peroxisome proliferator-activated receptor-gamma. *J. Exp. Med.* 201 1205–1215. 10.1084/jem.20041948 15824083 PMC2213148

[B34] Serra D. Almeida L. M. Dinis T. C. (2016). Anti-inflammatory protection afforded by cyanidin-3-glucoside and resveratrol in human intestinal cells via Nrf2 and PPAR-γ: Comparison with 5-aminosalicylic acid. *Chem. Biol. Interact.* 260 102–109. 10.1016/j.cbi.2016.11.003 27818126

[B35] Sofia M. A. Rubin D. T. (2016). Current approaches for optimizing the benefit of biologic therapy in ulcerative colitis. *Therap. Adv. Gastroenterol.* 9 548–559. 10.1177/1756283X16643242 27366223 PMC4913335

[B36] Su M. Cao J. Huang J. Liu S. Im D. S. Yoo J. W. (2017). The in vitro and in vivo anti-inflammatory effects of a phthalimide PPAR-γ agonist. *Mar. Drugs* 15:E7. 10.3390/md15010007 28054961 PMC5295227

[B37] Ungaro R. Mehandru S. Allen P. B. Peyrin-Biroulet L. Colombel J. F. (2017). Ulcerative colitis. *Lancet* 389 1756–1770. 10.1016/S0140-6736(16)32126-2 27914657 PMC6487890

[B38] Uwada J. Yazawa T. Islam M. T. Khan M. R. I. Krug S. M. Fromm M. (2017). Activation of muscarinic receptors prevents TNF-α-mediated intestinal epithelial barrier disruption through p38 MAPK. *Cell. Signal.* 35 188–196. 10.1016/j.cellsig.2017.04.007 28412413

[B39] Wang M. Xiu L. Diao J. Wei L. Sun J. (2015). Sparstolonin B inhibits lipopolysaccharide-induced inflammation in 3T3-L1 adipocytes. *Eur. J. Pharmacol.* 769 79–85. 10.1016/j.ejphar.2015.10.050 26522926

[B40] Weidner C. de Groot J. C. Prasad A. Freiwald A. Quedenau C. Kliem M. (2012). Amorfrutins are potent antidiabetic dietary natural products. *Proc. Natl. Acad. Sci. U.S.A.* 109 7257–7262. 10.1073/pnas.1116971109 22509006 PMC3358853

[B41] Wu P. Guo Y. Jia F. Wang X. (2015). The effects of Armillarisin A on serum IL-1β and IL-4 and in treating ulcerative colitis. *Cell Biochem. Biophys.* 72 103–106. 10.1007/s12013-014-0413-7 25420534 PMC4457911

[B42] Xu X. Wang Y. Wei Z. Wei W. Zhao P. Tong B. (2017). Madecassic acid, the contributor to the anti-colitis effect of madecassoside, enhances the shift of Th17 toward Treg cells via the PPARγ/AMPK/ACC1 pathway. *Cell Death Dis.* 8:e2723. 10.1038/cddis.2017.150 28358365 PMC5386545

[B43] Yang S. Yu Z. Wang L. Yuan T. Wang X. Zhang X. (2017). The natural product bergenin ameliorates lipopolysaccharide-induced acute lung injury by inhibiting NF-kappaB activation. *J. Ethnopharmacol.* 200 147–155. 10.1016/j.jep.2017.02.013 28192201

[B44] Zhang J. Zhang Y. Xiao F. Liu Y. Wang J. Gao H. (2016). The peroxisome proliferator-activated receptorγagonist pioglitazone prevents NF-κB activation in cisplatin nephrotoxicity through the reduction of p65 acetylation via the AMPK-SIRT1/p300 pathway. *Biochem. Pharmacol.* 101 100–111. 10.1016/j.bcp.2015.11.027 26673543

