# Supplementary figures and images for: Bergenin, Acting as an Agonist of PPARγ, Ameliorates Experimental Colitis in Mice through Improving Expression of SIRT1, and Therefore Inhibiting NF-κB-Mediated Macrophage Activation
Source: Front Pharmacol. 2018 Jan 12;8:981. doi: 10.3389/fphar.2017.00981 (PMC5770370; doi:10.3389/fphar.2017.00981)

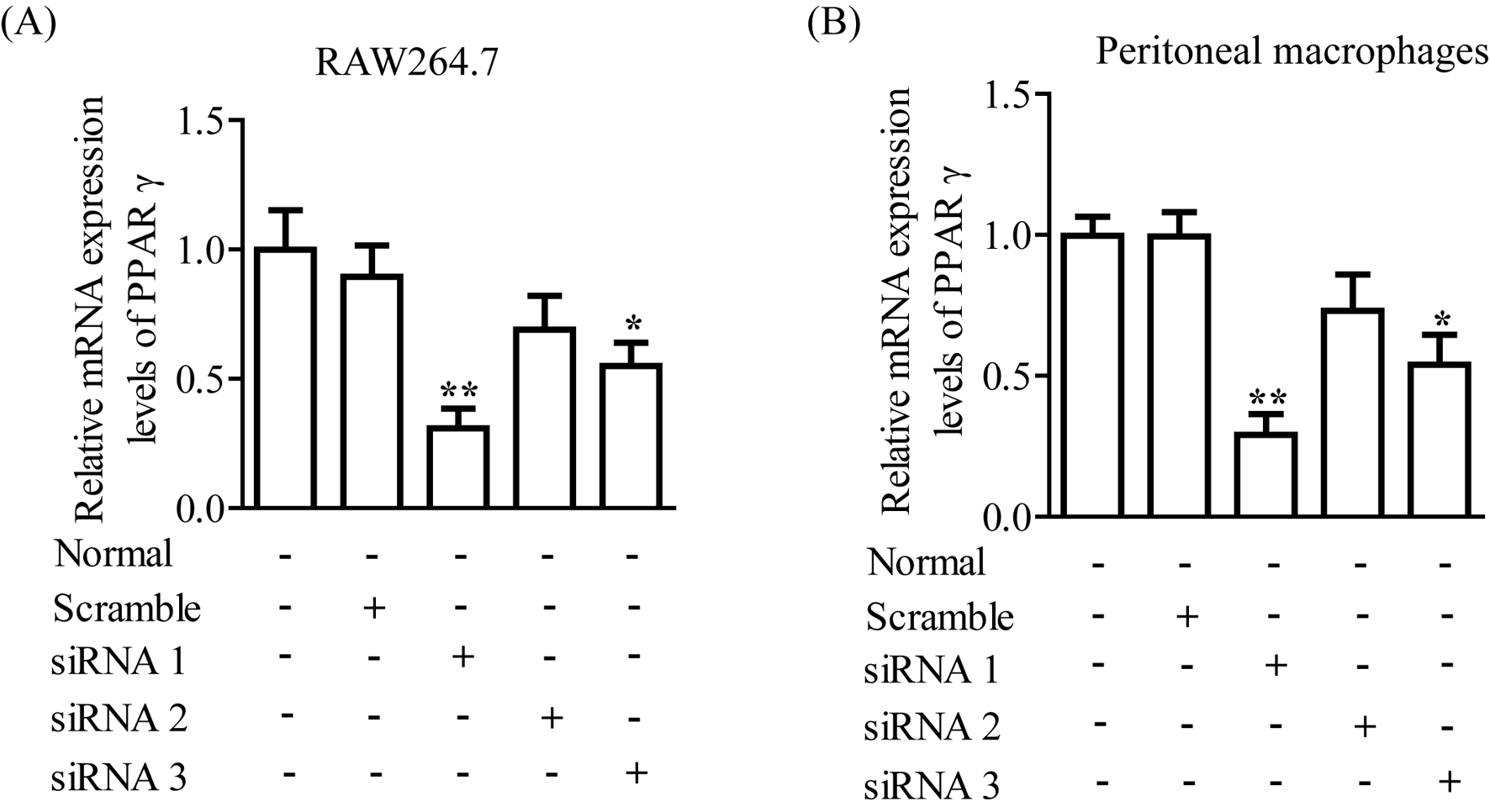

Supplement: FIGURE S1 — Effect of siPPAR-γ 1–3 on mRNA expression of PPAR-γ in (A) RAW264.7 cells and (B) peritoneal macrophages. Peritoneal macrophages and RAW264.7 cells were transfected with siPPARγ 1–3 for 24 h, and mRNA expression of PPAR-γ was detected by using Q-PCR assay. ∗p < 0.05 and ∗∗p < 0.01 vs. the group without any treatment. [file Image_1.TIF]
